# Unraveling complex relationships between COVID-19 risk factors using machine learning based models for predicting mortality of hospitalized patients and identification of high-risk group: a large retrospective study

**DOI:** 10.3389/fmed.2023.1170331

**Published:** 2023-05-04

**Authors:** Mohammad Mehdi Banoei, Haniyeh Rafiepoor, Kazem Zendehdel, Monireh Sadat Seyyedsalehi, Azin Nahvijou, Farshad Allameh, Saeid Amanpour

**Affiliations:** ^1^Department of Biological Sciences, University of Calgary, Calgary, AB, Canada; ^2^Cancer Biology Research Center, Cancer Institute, Tehran University of Medical Sciences, Tehran, Iran; ^3^Cancer Research Center, Cancer Institute, Tehran University of Medical Sciences, Tehran, Iran; ^4^Department of Medical and Surgical Sciences, University of Bologna, Bologna, Italy; ^5^Gastroenterology Ward, Imam Khomeini Hospital Complex (IKHC), Tehran University of Medical Sciences, Tehran, Iran

**Keywords:** COVID-19, prediction model, machine learning, COVID-19 risk factors, clustering COVID-19 patients

## Abstract

**Background:**

At the end of 2019, the coronavirus disease 2019 (COVID-19) pandemic increased the hospital burden of COVID-19 caused by the SARS-Cov-2 and became the most significant health challenge for nations worldwide. The severity and high mortality of COVID-19 have been correlated with various demographic characteristics and clinical manifestations. Prediction of mortality rate, identification of risk factors, and classification of patients played a crucial role in managing COVID-19 patients. Our purpose was to develop machine learning (ML)-based models for the prediction of mortality and severity among patients with COVID-19. Identifying the most important predictors and unraveling their relationships by classification of patients to the low-, moderate- and high-risk groups might guide prioritizing treatment decisions and a better understanding of interactions between factors. A detailed evaluation of patient data is believed to be important since COVID-19 resurgence is underway in many countries.

**Results:**

The findings of this study revealed that the ML-based statistically inspired modification of the partial least square (SIMPLS) method could predict the in-hospital mortality among COVID-19 patients. The prediction model was developed using 19 predictors including clinical variables, comorbidities, and blood markers with moderate predictability (*Q*^2^ = 0.24) to separate survivors and non-survivors. Oxygen saturation level, loss of consciousness, and chronic kidney disease (CKD) were the top mortality predictors. Correlation analysis showed different correlation patterns among predictors for each non-survivor and survivor cohort separately. The main prediction model was verified using other ML-based analyses with a high area under the curve (AUC) (0.81−0.93) and specificity (0.94−0.99). The obtained data revealed that the mortality prediction model can be different for males and females with diverse predictors. Patients were classified into four clusters of mortality risk and identified the patients at the highest risk of mortality, which accentuated the most significant predictors correlating with mortality.

**Conclusion:**

An ML model for predicting mortality among hospitalized COVID-19 patients was developed considering the interactions between factors that may reduce the complexity of clinical decision-making processes. The most predictive factors related to patient mortality were identified by assessing and classifying patients into different groups based on their sex and mortality risk (low-, moderate-, and high-risk groups).

## Introduction

The coronavirus disease 2019 (COVID-19) pandemic has affected more than 600 million individuals in more than 220 countries and regions, with more than 6.5 million deaths till 21 August, 2022 (1). The approximate basic production number is 3.15 with a 95% CI (2.41−3.90), while the estimated case fatality ratio is 2.72% with 95% CI (1.29−4.16%) (2). COVID-19 disease can manifest with a wide range of clinical features ranging from no symptoms to multi-organ failure (3). Although Severe acute respiratory syndrome coronavirus 2 (SARS-CoV-2) mainly affects the lungs, cardiovascular, neurological, renal, and vascular complications can also contribute to mortality (4). Accurate prognostication of clinical outcomes in this patient population can be challenging due to the high variability in disease severity; however, it is still essential considering the need for effective triage and efficient allocation of limited resources (i.e., beds and ventilators). Therefore, identification of high-risk patients and recognition of mortality predictors could possibly allow to offer more targeted approaches and better allocate resources. The identification of contributing factors would allow for applying targeted strategies in patients with the highest mortality risk. Patients’ accurate history, clinical signs, fever and oxygen saturation measurements, blood cell counts (CBCs), other laboratory findings, computed tomography (CT) scan imaging, and real-time reverse-transcription polymerase chain reaction (RT-PCR) test are included in prognostic and diagnostic criteria (5, 6). According to the previous studies, some laboratory findings such as lymphopenia, neutropenia, increased alanine aminotransferase (ALT), aspartate aminotransferase (AST), lactate dehydrogenase (LDH), high-sensitivity C-reactive protein (hs-CRP), and some clinical signs such as myalgia and shortness of breath had a relationship with an increased mortality and could also be considered as risk factors for COVID-19 mortality (7, 8). In addition, underlying diseases such as diabetes, cardiovascular disease (CVD), chronic pulmonary disease (CPD), chronic liver disease (CLD), chronic kidney disease (CKD), rheumatic diseases, cerebrovascular diseases, cancers, immunodeficiency diseases, hypertension, risk factors such as high body mass index (BMI), hyperlipidemia, and history of smoking are also among the factors that can lead to more severe forms of the disease (9, 10). In this regard, developing a mortality prediction model using artificial intelligence approaches such as deep learning for radiography and computed tomography (CT) image analysis (11–16) and multivariable analysis could be of tremendous value. To obtain invaluable knowledge from the high-dimensional data effectively, projection-based machine learning (ML) methods such as statistically inspired modification of the partial least square (SIMPLS), random forest (RF), support vector machine (SVM), and artificial neural network (ANN) have been increasingly utilized (17–19). The ML methods are discriminated from conventional statistical methods such as logistic regression, Cox regression, generalized additive models, and least-square linear regression method by presenting accurate predictions, enjoying flexibility and scalability, and finding the relationships between variables and internal validity of ML methods using large datasets (20). In contrast to conventional prediction models that only use input data, artificial intelligence-oriented models are more capable of dealing with continuous data involving unpredictability and uncertainty, which in turn lead to integrated results (21). This property can help healthcare professionals use ML-based methods in clinical settings and understand physiological and biological processes to fight human diseases and global pandemics such as COVID-19 (22).

In the current study, an ML-based statistical method was applied to predict mortality among COVID-19 patients and identified the complex relationship between predictors in clinical practice. The clustering methods were also used to categorize the patients based on sex and mortality risk (low-, moderate-, and high-risk groups). To achieve this aim, 82 variables including clinical data, comorbidities, and biochemical data were used in a large cohort of COVID-19 patients. Understanding the potential predictors of outcome in COVID-19 patients, including oxygen saturation <88, loss of consciousness, and chronic kidney disease as the top three mortality predictors, is vital to make an appropriate clinical decision and improve the healthcare system to provide better disease management services.

**TABLE 1:** Distribution of patients’ demographics, clinical variables, comorbidities, and blood analytes and cells between COVID-19 non-survivors and survivors.

Variables	Dead (*n* = 305) number (%)	Alive (*n* = 1439) number (%)	*P*- value
Male	200 (65.5%)	831 (57.7%)	<0.0001
Age (years) M ± SD	57.69 ± 18.23	57.96 ± 16.59	0.805*
ICU admission	213 (69.8%)	147 (10.2%)	<0.0001
ICU length of stay	5.67 ± 9.14	0.61 ± 3.24	<0.0001
BMI M ± SD	23.01 ± 3.70	24.25 ± 10.80	0.048
Smoking and alcohol and drug	45 (14.7%)	131 (0.09%)	<0.0001
Chronic pulmonary disease	42 (13.7%)	138 (0.09%)	<0.0001
Hypertension	211 (69.1%)	670 (46.5%)	<0.0001
Hypotension	4 (1.3%)	(0.2%)	<0.0001
Cerebrovascular accident	20 (6.5%)	41 (2.8%)	<0.0001
Cancer	43 (12.2%)	103 (7.1%)	<0.0001
Cardiovascular disease	115 (37.7%)	338 (23.4%)	<0.0001
Chronic kidney disease	85 (27.8%)	111 (7.7%)	<0.0001
Chronic liver disease	10 (3.2%)	24 (1.6%)	0.001
Neurological disease	23 (7.5%)	64 (4.4%)	<0.0001
Immunodeficiency disease	3 (0.9%)	12 (0.8%)	<0.0001
Rheumatic disease	20 (6.5%)	42 (2.9%)	<0.0001
Gastrointestinal ulcer	4 (1.3%)	23 (1.5%)	0.002
Hemiplegia	2 (0.6%)	10 (0.6%)	0.002
HIV	4 (1.3%)	3 (0.2%)	<0.001
Diabetes	124 (40.6%)	478 (33.2%)	<0.0001
Hyperthyroidism	19 (6.2%)	110 (7.6%)	0.001
Transplantation	4 (1.3%)	16 (1.1%)	0.002
Loss of consciousness	47 (15.4%)	18 (1.2%)	<0.0001
Heart abnormal findings	110 (36%)	210 (14.5%)	<0.0001
Weight loss	5 (1.6%)	16 (1.1%)	0.002
Wet cough	63 (20.6%)	277 (19.2%)	0.001
Trembling	95 (31.1%)	664 (46.1%)	<0.0001
Sweating	22 (7.2%)	172 (11.9%)	<0.0001
Sputum	32 (10.4%)	92 (6.3%)	<0.0001
Rhinorrhea	9 (2.9%)	34 (2.3%)	0.002
Muscle pain myalgia	120 (39.3%)	774 (53.7%)	<0.0001
Loss of taste	3 (0.9%)	80 (5%)	<0.0001
Loss of smell	3 (0.9%)	97 (6.7%)	<0.0001
Limb edema	21 (6.8%)	32 (2.2%)	<0.0001
Joint pain arthralgia	8 (2.5%)	56 (3.8%)	0.001
Hemoptysis	16 (5.2%)	58 (4%)	0.001
Fatigue	13 (4.2%)	49 (3.4%)	0.002
Epigastric	27 (8.8%)	146 (10.1%)	0.002
Dizziness	16 (5.2%)	125 (8.6%)	<0.0001
Diarrhea	33 (10.8%)	246 (17%)	<0.0001
Chest pain	25 (5.5%)	224 (15.5%)	<0.0001
Cardiac arrhythmia	3 (0.9%)	13 (0.9%)	0.002
Temperature	37.19 ± 0.84	37.19 ± 0.87	0.993*
Systolic blood pressure M ± SD	122.22 ± 22.30	121.96 ± 17.30	0.821*
Diastolic blood pressure M ± SD	75.39 ± 13.05	78.96 ± 33.82	0.069*
Heart rate M ± SD	96.10 ± 19.9	94.35 ± 37.3	0.427*
Respiratory rate M ± SD	21.46 ± 7.82	20.45 ± 7.52	0.036
Oxygen saturation (percent) M ± SD	83.60 ± 11.61	90.76 ± 6.23	<0.0001
Hemoglobin (HB) M ± SD	13.00 ± 2.46	13.48 ± 2.18	0.001
Fasting blood sugar (FBS) M ± SD	132.47 ± 91.24	98.95 ± 54.31	<0.0001
ESR M ± SD	56.26 ± 40.31	48.14 ± 38.59	0.001
Ferritin (I) M ± SD	589.47 ± 867.36	295.29 ± 432.65	<0.0001
Urea M ± SD	55.87 ± 63.83	34.70 ± 28.27	<0.0001
pHM ± SD	7.39 ± 0.03	7.40 ± 0.01	0.004
D-dimer M ± SD	1,073 ± 2,217	1490 ± 25,067	0.772*
Creatinine M ± SD	1.62 ± 1.30	1.20 ± 0.80	<0.0001
BSM ± SD	108.60 ± 106.26	71.05 ± 91.54	<0.0.0001
Albumin M ± SD	5.12 ± 13.99	5.31 ± 44.73	0.940
ALT M ± SD	49.06 ± 106.94	35.00 ± 30.51	<0.0.0001
AST M ± SD	34.16 ± 123.72	24.71 ± 21.81	0.007
ALP M ± SD	94.39 ± 97.13	83.55 ± 37.41	0.001
LDH M ± SD	185.43 ± 161.88	193.95 ± 333.58	0.191*
BNP M ± SD	399.95 ± 2783.98	37.86 ± 524.14	<0.0001
Troponin M ± SD	16.54 ± 131.92	2.44 ± 65.51	0.006
CPK M ± SD	126.01 ± 212.49	100.17 ± 49.09	<0.0001
Direct bilirubin M ± SD	0.30 ± 1.13	0.21 ± 0.07	0.002
Total bilirubin M ± SD	0.82 ± 1.52	0.66 ± 0.17	<0.0001
Calcium (Ca) M ± SD	8.43 ± 1.05	8.83 ± 0.86	<0.0001
Sodium (Na) M ± SD	138.41 ± 4.63	138.90 ± 3.79	0.049
Potassium (K) M ± SD	4.27 ± 0.67	4.18 ± 0.55	0.008
Phosphorus (P) M ± SD	3.75 ± 1.22	3.64 ± 0.81	0.057*
Magnesium (Mg) M ± SD	2.18 ± 0.49	2.15 ± 0.54	0.314*
WBC M ± SD	7,925 ± 8,710	29,268 ± 45,967	0.416*
Neutrophil M ± SD	7,404 ± 6,785	23,362 ± 2,58,760	0.437*
Lymphocyte M ± SD	1,519 ± 1,341	4,091 ± 52,326	0.391*
PLT M ± SD	2,32,662 ± 10,4536	2,41,032 ± 1,00,840	0.191*

*non-significant.

## Materials and methods

### Data collection

The present retrospective study was conducted by the Tehran University of Medical Science (TUMS) in Imam Khomeini Hospital Complex (IKHC) following the study of Allameh et al. (22, 23). A total of 1,743 RT-PCR confirmed COVID-19 cases were enrolled in the study. Overall, 82 variables including clinical data, comorbidities, and biochemical data were collected at the hospital admission, intensive care unit (ICU) admission, and hospital discharge time. Moreover, 42 binary and 40 continuous variables were attended to in this study.

The initial clinical data element collected during the patient encounter was considered the presentation data. These data included (a) demographic variables such as age, sex, and BMI, (b) comorbidities including diabetes, CVD, hypertension, CKD, neurological diseases, and immunodeficiency disease, and (c) clinical variables consisting of oxygen saturation level, fever, loss of smell, loss of taste, and wet cough. To capture much more complicated relationships and facilitate interpretations and presentations of results, continuous variables were converted to dichotomous variables using predictive partition analysis. Of course, continuous variables were also used to create a prediction model. Data underwent filtration when the variables were missed or not measured in more than 85% of cases. The handling of the missing data was performed using the mean imputation method in all data analysis and prediction models.

### Statistical analysis

A pro version of JMP (JMP^®^ v. 16.1.0/SAS Institute Inc.) statistical discovery software was used for the data analysis. The SIMPLS analysis was applied to create prediction models using patients’ characteristics, clinical symptoms, comorbidities, and biochemical data collected at the hospital admission time. As an algorithm of PLS (a linear machine learning method) (24, 25). SIMPLS is an alternative algorithm for partial least square (PLS) regression that has been proposed to calculate the PLS factors by maximizing covariance in the linear approach of the original variable’s combination. SIMPLS is able to find the score vectors of multiple Xs and Ys variables. The advantage of SIMPLS is to compute the factors from the original (centered) data. The R weights obtained by SIMPLS are associated with a simpler interpretation compared to W weights. SIMPLS is a faster method since the algorithm does not need to break down the X matrix. SIMPLS could be similar to PLS1 in terms of univariate Y, but it is different from PLS2 regarding multivariate Y in that the covariance criterion is maximized by SIMPLS (25).

Statistically inspired modification of the partial least square was performed using training and validation sets. The validation set comprised 519 subjects that were automatically and randomly created based on approximately 30% of 1,743 hospitalized COVID-19 patients. Initially, the prediction model was created using all variables, and the best prediction model was obtained using the variable importance in projection (VIP) score of greater than 1.0. The VIP is defined as a weighted sum of squares of the variable’s weights and shows the contribution of variables to predict and characterize the factors in the model (26). The statistics Q^2^ (goodness of prediction) and R^2^Y (goodness of variation) were obtained by SIMPLS using the leave-one-out cross-validation (CV) procedure. The CV, known as internal validation, constructed Q^2^ and R^2^Y based on the training set and validated them using the validation set. The prediction model determines the number of factors to be included in the model. The best prediction model was selected when Q^2^ had the maximum value and did not start decreasing. In addition, R^2^Y was higher than Q^2^, which prevented overfitting. Moreover, the partition analysis was used to create a decision tree of the data partition according to a relationship between the outcome and predictors. The data were partitioned into training and validation sets. The partition algorithm searched all possible splits of predictors to best predict the response. The most differentiating clinical predictors obtained by SIMPLS used the partition analysis. The predictive partition analysis was applied for categorizing continuous variables by obtaining the best cutoff point for each variable such as age, BMI, clinical symptoms [heart rate (HR) and respiratory rate (RR)], and blood biochemical data. The partition prediction algorithm found all possible splits of the variables to predict the response. As there might be more than one cutoff point for each variable, the first cutoff points were selected for the best split. Furthermore, the partition analysis was used to obtain cutting values for either continuous or categorical (nominal or ordinal) variables such as age, HR, RR, and BMI. Principal component analysis (PCA) was used to present the differentiation between survivors and non-survivors in an unsupervised manner using all variables. PCA was also used to cluster subgroups using only the most differentiating variables obtained from SIMPLS. PCA analysis was carried out in two steps. The first step used all variables to find outliers and trends, while the second step used the most differentiating predictors obtained from SIMPLS method. Latent class analysis (LCA) was applied to identify the COVID-19 patients at the highest risk of mortality by clustering patients into subgroups with high, moderate, and low mortality rates. All continuous variables were normalized, transformed, and scaled to be used independently or in combination with binary data for predicting the mortality rate. The model screening was performed to verify the final prediction model by providing a summary table using the other ML methods such as SVM, K-nearest neighbor (KNN), generalized regression lasso (GRL), boosting neural networks (BNN), and random forest (RR). Model screening also helps to find an efficient workflow as well as compare and explore datasets for the best predictive model.

## Results

### Patients’ characteristics

A total of 2,498 patients registered in the COVID-19 registry based on their hospitalization between 20 February, 2020, and 27 October, 2020, were included in the analyses. These patients had positive SARS-CoV-2 RT-PCR. A total of 1,743 COVID-19 patients were enrolled in the study with 17.4% in-hospital mortality (*n* = 305). Table 1 shows the demographic characteristics, comorbidities, clinical symptoms, and blood biochemical concentrations of COVID-19 survivors and non-survivors that were admitted to the hospital. Table 1 indicates that most comorbidities and clinical symptoms were significantly different between non-survivors and survivors. Among the analyzed blood concentrations, only D-dimer and LDH were not significantly different between COVID-19 survivors and non-survivors, and none of the blood cells were significantly different between the two groups.

### Predicting hospital mortality using machine learning-based model

**FIGURE 1:**
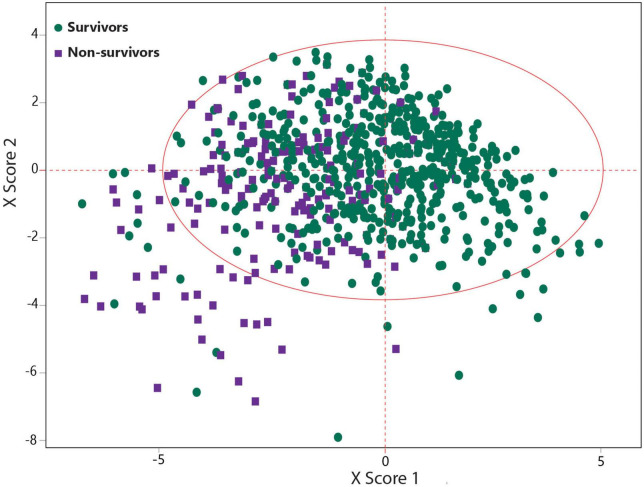
Statistically inspired modification of the partial least square (SIMPLS)-based scatter plot indicating a good separation between COVID-19 survivors and non-survivors.

Patients’ demographics, clinical symptoms, comorbidities, and blood analytes were used for predicting the hospital mortality using the SIMPLS as an ML-based multivariate data analysis model (Figure 1). The best prediction model was obtained by SIMPLS using the most differentiating variables with VIP > 0.8 (26). The prediction of mortality was determined based on the 1,224 and 519 COVID-19 patients in the training and validation sets, respectively. The best model to predict the mortality had moderate predictability (*Q*^2^ = 0.259) with the variability of *R*^2^ = 0.267 using 19 most differentiating predictors that contributed to the prediction. Table 2 presents 19 predictors ordered by their importance in the model. In this regard, oxygen saturation <88% was the most important variable to predict the COVID-19 mortality and was followed by loss of consciousness, CKD, heart abnormal findings, hypertension, and age >65.

**TABLE 2:** The most differentiating predictors and their importance in the projection (VIP) scores for the predicting the mortality outcome.

	Variables	VIP
1	Oxygen saturation < 88	3.21
2	Loss of consciousness	2.49
3	Chronic kidney disease	2.32
4	Heart abnormal finding	2.15
5	Hypertension	2.04
6	Age > 65	1.87
7	Cardiovascular disease	1.47
8	Trembling	1.24
9	Muscle pain myalgia	1.22
10	Limb edema	1.12
11	Sputum	0.84
12	Rheumatological disease	0.96
13	Loss of smell	0.94
14	Cancers	0.94
15	Diabetes	0.98
16	ALT = 13	0.92
17	Potassium = 4	0.84
18	Loss of taste	0.83
19	BMI = 24.8	0.83

Although most of variables were found to be significant predictors between non-survivors and survivors (Table 1) using univariate analysis, the multivariate prediction model revealed that 19 variables ordered by their importance in the model could be the best and most predictable variables for predicting the mortality.

**FIGURE 2:**
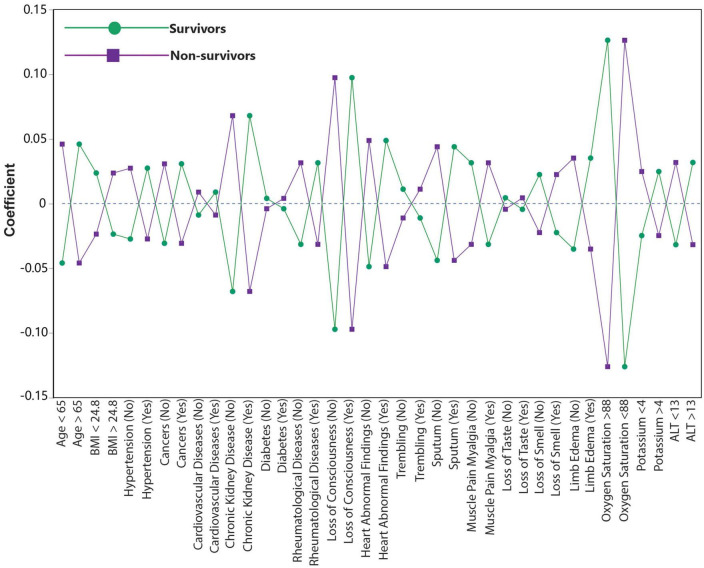
Coefficient plot shows the relative correlation of 19 most differentiating variables to predict mortality. Loss of consciousness, oxygen saturation < 88 and chronic kidney disease shows the highest relative correlation with mortality.

The coefficient plot showed the positive correlation of age >65, presence of hypertension, cancer, CVD, CKD, rheumatic disease, heart abnormal findings, sputum, limb edema, loss of consciousness, oxygen saturation <88%, potassium >4, and ALT >13 with the mortality among COVID-19 patients. Astonishingly, BMI > 24.8, diabetes, trembling, muscle pain myalgia, loss of taste, and loss of smell were negatively correlated with the mortality. The coefficient plot illustrated the correlation between predictors and mortality (Figure 2).

Principal component analysis-based correlation analysis showed a high positive correlation between age >65, hypertension, CVD, CKD, and heart abnormal findings. Diabetes was correlated with the above-mentioned predictors, with the exception of heart abnormal findings. Trembling, muscle pain myalgia, and loss of taste and smell were negatively correlated with age > 65, hypertension, CVD, CKD, and heart abnormal findings. Interestingly, oxygen saturation < 88% showed a relatively high correlation with only age > 65, hypertension, and heart abnormal findings. Fascinatingly, although BMI > 24.8 was not correlated with comorbidities and oxygen saturation, it somehow had a positive correlation with clinical symptoms (Table 3).

**TABLE 3:** The PCA correlation table shows the correlation between the most differentiating predictors.

	Age > 65	BMI > 24.8	Hypertension	Cancers	Cardiovascular diseases	Chronic kidney disease	Diabetes	Rheumatological diseases	Loss of consciousness	Heart abnormal findings	Trembling	Sputum	Muscle pain myalgia	Loss of taste	Loss of smell	Limb edema	Oxygen saturation < 88	Potassium > 4	ALT > 13
Age > 65	1	-0.08	0.303	0.021	0.263	0.075	0.16	0.023	0.022	0.111	-0.11	0.006	-0.13	-0.08	-0.1	0.007	-0.12	0.02	-0.01
BMI > 24.8	-0.08	1	-0.01	-0.08	-0.01	0.01	0.012	0.006	0.009	0.09	0.121	0.042	0.087	0.085	0.045	-0.02	0.053	-0.01	0.061
Hypertension	0.303	-0.01	1	-0.02	0.335	0.178	0.287	0.035	0.08	0.247	-0.1	0.015	-0.07	-0.05	0.09	0.075	-0.1	0.04	0.016
Cancers	0.021	-0.08	-0.02	1	-0.03	0.023	0.003	-0	0.061	0.092	-0.08	0.021	-0.1	-0.03	-0.03	0.055	0.007	0.005	-0.01
Cardiovascular diseases	0.263	-0.01	0.335	-0.03	1	0.145	0.158	0.02	0.028	0.202	-0.1	0.014	-0.06	-0.04	-0.05	0.093	-0.07	0.022	0.018
Chronic kidney disease	0.075	0.01	0.178	0.023	0.145	1	0.131	0.059	0.15	0.164	-0.01	0.029	-0.02	-0.05	-0.04	0.096	-0.05	0.066	0.087
Diabetes	0.16	0.012	0.287	0.003	0.158	0.131	1	0.023	0.042	0.045	-0.06	0.024	-0.02	0.002	-0.02	0.019	-0.05	-0.03	-0.01
Rheumatological diseases	0.023	0.006	0.035	-0	0.02	0.059	0.023	1	0.011	0.013	-0.01	0.007	-0.02	-0.03	-0.03	0.056	-0.03	-0.01	0.001
Loss of consciousness	0.022	0.009	0.08	0.061	0.028	0.15	0.042	0.011	1	0.063	-0.02	0.04	-0.04	0.013	-0.01	0.018	-0.1	-0	-0.01
Heart abnormal findings	0.111	0.09	0.247	0.092	0.202	0.164	0.045	0.013	0.063	1	-0.11	-0.04	-0.12	-0.02	-0.03	0.115	-0.11	0.054	0.041
Trembling	-0.11	0.121	-0.1	-0.08	-0.1	-0.01	-0.06	-0.01	-0.02	-0.11	1	0.018	0.219	0.097	0.102	-0.03	0.073	-0	-0.04
Sputum	0.006	0.042	0.015	0.021	0.014	0.029	0.024	0.007	0.04	-0.04	0.018	1	0.06	0.074	0.066	-0.04	0.003	0.032	-0.04
Muscle aain myalgia	-0.13	0.087	-0.07	-0.1	-0.06	-0.02	-0.02	-0.02	-0.04	-0.12	0.219	0.06	1	0.11	0.097	-0.06	0.012	-0	-0
Loss of taste	-0.08	0.085	-0.05	-0.03	-0.04	-0.05	0.002	-0.03	0.013	-0.02	0.097	0.074	0.11	1	0.64	0.007	0.075	-0.04	-0.02
Loss of smell	-0.1	0.045	0.09	-0.03	-0.05	-0.04	-0.02	-0.03	-0.01	-0.03	0.102	0.066	0.097	0.64	1	-0	0.072	-0.03	-0.01
Limb edema	0.007	-0.02	0.075	0.055	0.093	0.096	0.019	0.056	0.018	0.115	-0.03	-0.04	-0.06	0.007	-0	1	-0.04	0.024	0.023
Oxygen saturation < 88	-0.12	0.053	-0.1	0.007	-0.07	-0.05	-0.05	-0.03	-0.1	-0.11	0.073	0.003	0.012	0.075	0.072	-0.04	1	-0.03	-0.04
Potassium > 4	0.02	-0.01	0.04	0.005	0.022	0.066	-0.03	-0.01	-0	0.054	-0	0.032	-0	-0.04	-0.03	0.024	-0.03	1	0.044
ALT > 13	-0.01	0.061	0.016	-0.01	0.018	0.087	-0.01	0.001	-0.01	0.041	-0.04	-0.04	-0	-0.02	-0.01	0.023	-0.04	0.044	1

Cardiovascular diseases, chronic kidney disease and hypertension have more correlation with other predictors. Positive correlations are shown in red while negative correlations are shown in blue. The intensity of the color is related to correlation coefficients.

**FIGURE 3:**
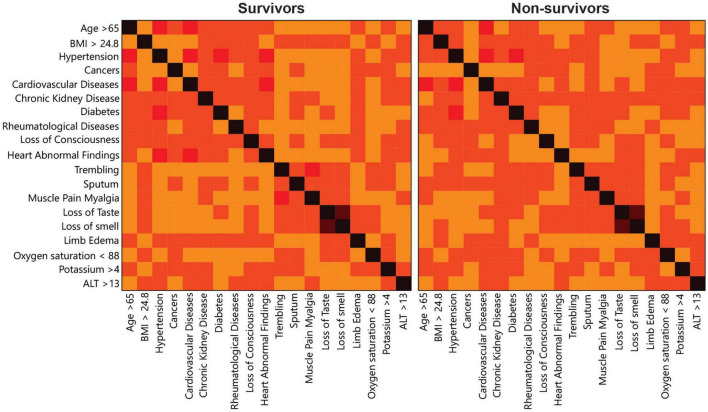
Multivariate correlation heat map clearly indicating a different pattern between survivors and non-survivors. Age > 65, BMI > 24.8 and hypertension as well as oxygen saturation < 88, cardiovascular disease and chronic kidney disease are more correlated among non-survivors than survivors.

Multivariate correlation analysis showed a different correlation pattern between the predictors for each of the non-survivor and survivor cohorts separately (Figure 3). According to Figure 3, the correlations between hypertension, heat abnormal findings, CVD, and age >65 discriminated non-survivors from survivors.

Model screening showed high AUCs (>0.80), high specificities (>90%), and good sensitivities (>70%) using the most differentiating predictors in other ML methods such as SVM, KNN, and GRL (Table 4).

**TABLE 4:** Model screening of prediction mortality COVID-19 patients shows a high AUC and specificity for most ML-based methods such as support vector machine, neural boosted and K Nearest Neighbors.

Method	*N*	Entropy R^2^	Misclassification rate	AUC	RASE	Generalized R^2^	Sensitivity	Specificity
**Training set**								
Bootstrap forest	1224	0.419	0.125	0.927	0.290	0.532	63	97
Boosted tree	1224	0.414	0.109	0.917	0.287	0.527	73	97
Neural boosted	1224	0.312	0.143	0.874	0.316	0.416	61	95
Nominal logistic	1224	0.287	0.147	0.866	0.322	0.386	66	96
Generalized regression lasso	1224	0.269	0.152	0.863	0.325	0.365	74	97
Support vector machines	1224	0.248	0.147	0.885	0.322	0.34	85	99
Decision tree	1224	0.234	0.158	0.82	0.334	0.323	71	96
Fit stepwise	1224	0.22	0.152	0.825	0.335	0.308		
K nearest neighbors	1224	0.152	0.168				80	98
**Validation set**								
Neural boosted	519	0.291	0.144	0.857	0.317	0.392	57	94
Fit stepwise	519	0.282	0.146	0.846	0.319	0.382		
Generalized regression lasso	519	0.258	0.152	0.844	0.325	0.353	72	97
Nominal logistic	519	0.240	0.152	0.840	0.328	0.331	61	94
Boosted tree	519	0.238	0.158	0.837	0.331	0.328	55	98
Decision tree	519	0.235	0.1541	0.8112	0.33513	0.3247	70	96
Support vector machines	519	0.2316	0.1387	0.8354	0.32522	0.3205	81	99
Bootstrap forest	519	0.1909	0.1638	0.8072	0.34281	0.2691	73	97
K nearest neighbors	519	0.0855	0.1734				83	96

### Mortality prediction model revealing the difference in the prognosis of mortality rate between male and female patients

Statistically inspired modification of the partial least square-based prediction models showed that the prediction of mortality was different between male (training set = 723, validation set = 308) and female (training set = 501, validation set = 211) patients. The best mortality prediction models used the most differentiating variables with VIP > 1.0 and revealed the predictabilities of *Q*^2^ = 0.243 and *Q*^2^ = 0.195 for males and female, respectively. Hence, the best models were obtained by 26 (VIP > 0.8) and 19 (VIP > 1.0) predictors among males and females, respectively, (Table 5). Remarkably, oxygen saturation < 88%, loss of consciousness, and CKD were the top important predictors for predicting the mortality among male and female patients. Nonetheless, unique mortality predictors among male patients indicated that muscle pain myalgia, loss of smell, creatine > 1.7, and ALY > 23 had a negative correlation with the mortality, while sputum, CPD, smoking/alcohol/drug history, urea > 49, and transplantation history had a positive correlation with the mortality. Unique mortality predictors among female patients showed that BMI > 24.8, chest pain, calcium > 9, and sweating had a negative correlation with the mortality, whereas rheumatic disease, CLD, and human immunodeficiency virus (HIV) had a positive correlation with the mortality. Interestingly, the correlation of trembling, Mg > 2.2, and loss of taste with the mortality was different between male and female patients. The male-specific model for predicting the mortality showed the differences in two overlapped trembling and loss of taste predictors with the general prediction model. These predictors had positive and negative correlations with mortality in the male-specific and general models, respectively. Model screening showed high AUCs > 0.89 and > 0.81 for models for men and women, respectively, with high specificities (>95%), and good sensitivities (>70%) for models of women than men using the most differentiating predictors in other ML methods such as SVM, KNN, and GRL (Supplementary Tables 1, 2).

**TABLE 5:** The most differentiating predictors and their importance in the projection (VIP) scores for the predicting the mortality outcome A: among men COVID-19 patients, B: among women COVID-19 patients.

	Predictors	VIP	Correlation with mortality
**(A)**			
1	Oxygen saturation < 88	3.45	Positive
2	Loss of consciousness	2.69	Positive
3	Chronic kidney disease	2.55	Positive
4	Hypertension	2.44	Positive
5	Heart abnormal findings	2.45	Positive
6	Age > 65	1.92	Positive
7	Cardiovascular disease	1.77	Positive
8	Muscle pain myalgia	1.51	Negative
9	ALT > 13	1.40	Positive
10	Sputum	1.41	Positive
11	Potassium > 4.0	1.11	Positive
12	Diarrhea	1.12	Negative
13	Total lung involvement	1.09	Positive
14	Diabetes	0.99	Negative
15	Trembling	1.03	Positive
16	Loss of sense of smell	0.96	Negative
17	Chronic pulmonary disease	0.94	Positive
18	Smoking alcohol drug history	0.95	Positive
19	Limb edema	0.91	Positive
20	Mg > 2.2	0.99	Negative
21	Cancers	0.91	Positive
22	Urea > 49	0.85	Positive
23	Loss of sense of taste	0.82	Positive
24	Creatine > 1.7	0.93	Negative
25	Transplantation	0.81	Positive
26	ALY > 23	0.93	Negative
**(B)**			
1	Oxygen saturation < 88	3.24	Positive
2	Loss of consciousness	2.89	Positive
3	Chronic kidney disease	2.27	Positive
4	Heart abnormal findings	2.05	Positive
5	Trembling	1.93	Negative
6	Hypertension	1.68	Positive
7	Age > 65	1.74	Positive
8	Rheumatological diseases	1.74	Positive
9	Limb edema	1.59	Positive
10	BMI > 24.8	1.38	Negative
11	Cardiovascular disease	1.34	Positive
12	Chronic liver disease	1.17	Positive
13	HIV	1.15	Positive
14	Chest pain	1.15	Negative
15	Cancers	1.09	Positive
16	Calcium > 9	1.01	Negative
17	Sweating	1.01	Negative
18	Mg > 2.2	1.00	Positive
19	Loss of sense of taste	1.02	Negative

The gray highlighted variable represents unique predictor between models of men and women.

### Classification of COVID-19 patients to low-, moderate-, and high-risk groups

**FIGURE 4:**
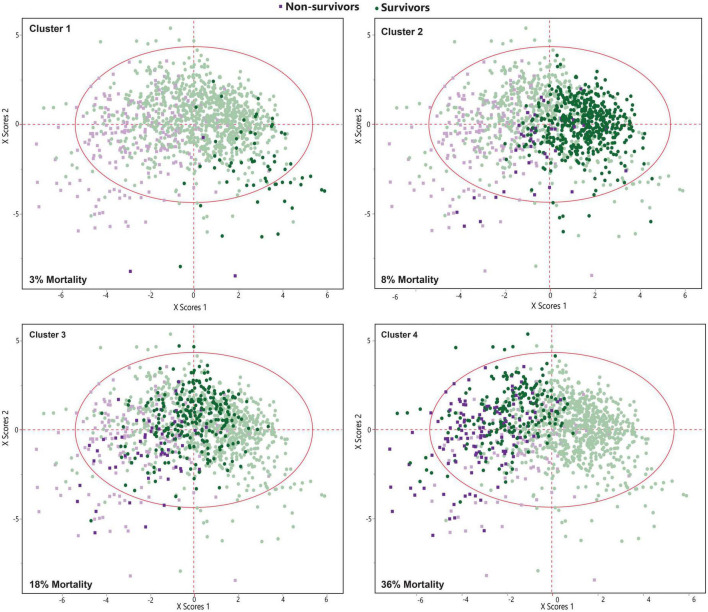
Principal component analysis (PCA) plot illustrates the Latent class analysis (LCA)-based clustering of COVID-19 patients. Clusters 3 and 4 are correlated with a higher mortality rate.

**FIGURE 5:**
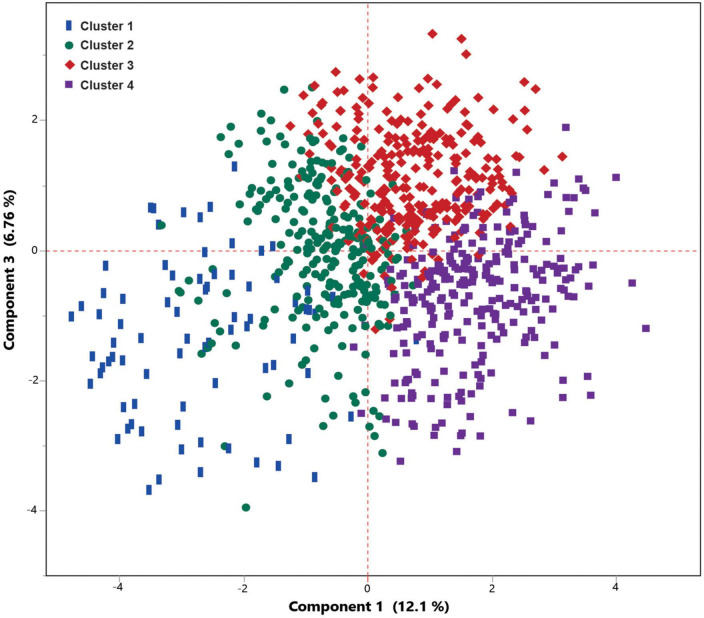
Principal component analysis (PCA) scatter plot shows a very good separation between four clusters obtained from Latent class analysis (LCA) analysis. Cluster 1 and 2 included the patients with a lower mortality risk, while clusters 3 included moderate risk of mortality, and cluster 4 included patients with higher mortality.

Latent class analysis (LCA) was applied to cluster the COVID-19 patients into low-, moderate-, and high-risk groups using 19 most differentiating predictors obtained from the SIMPLS prediction models. The best model was obtained by 4 clusters using LCA among COVID-19 patients. LCA-based clusters showed 3%, 8% (low risk), 18% (moderate risk), and 36% (high risk) of the mortality rate due to COVID-19 for the clusters 1, 2, 3, and 4, respectively, (Figures 4, 5). Table 6 shows the coefficient contribution of each variable in different LCA-based clustering groups. The mentioned table indicates that cluster 1 with the lowest mortality rate (3%) was apparently characterized by age <65 and the presence of trembling, muscle pain myalgia, and loss of taste and smell. On the other hand, the presence of hypertension, CKD, and diabetes was highly correlated with cluster 4 with the highest mortality rate (36%). Remarkably, cluster 3 with the second highest mortality rate (18%) was similar to cluster 4; however, it was well-characterized by a higher correlation of age >65, cancers, CVD, heart abnormal findings, oxygen saturation <88%, and potassium >4.

**TABLE 6:** The conditional probabilities for each cluster are presented for each response category of 19 variables in the analysis.

Predictors	Category	Cluster 1	Cluster 2	Cluster 3	Cluster 4
Age > 65	No	0.850	0.834	0.376	0.482
Age > 65	Yes	0.150	0.166	0.624	0.518
BMI > 24.8	No	0.651	0.800	0.939	0.697
BMI > 24.8	Yes	0.349	0.200	0.061	0.303
Hypertension	No	0.646	0.879	0.202	0.132
Hypertension	Yes	0.354	0.121	0.798	0.868
Cancers	No	0.955	0.925	0.855	0.955
Cancers	Yes	0.045	0.075	0.145	0.045
Cardiovascular disease	No	0.856	0.956	0.529	0.577
Cardiovascular disease	Yes	0.144	0.044	0.471	0.424
Chronic kidney disease	No	0.964	0.976	0.812	0.803
Chronic kidney disease	Yes	0.036	0.024	0.188	0.197
Diabetes	No	0.668	0.837	0.556	0.454
Diabetes	Yes	0.332	0.163	0.444	0.546
Rheumatological diseases	No	0.988	0.969	0.959	0.956
Rheumatological diseases	Yes	0.012	0.031	0.041	0.044
Loss of consciousness	No	0.955	0.988	0.922	0.963
Loss of consciousness	Yes	0.045	0.012	0.078	0.037
Heart abnormal findings	No	0.878	0.950	0.549	0.856
Heart abnormal findings	Yes	0.122	0.050	0.451	0.144
Trembling	No	0.300	0.494	0.854	0.441
Trembling	Yes	0.700	0.506	0.146	0.559
Sputum	No	0.816	0.947	0.959	0.892
Sputum	Yes	0.184	0.053	0.041	0.108
Muscle pain myalgia	No	0.196	0.442	0.783	0.320
Muscle pain myalgia	Yes	0.804	0.558	0.217	0.680
Loss of taste	No	0.164	0.999	0.992	0.999
Loss of taste	Yes	0.836	0.001	0.008	0.001
Loss of smell	No	0.251	0.971	0.989	0.993
Loss of smell	Yes	0.749	0.030	0.011	0.007
Limb edema	No	0.965	0.991	0.921	0.983
Limb edema	Yes	0.035	0.009	0.079	0.017
Oxygen saturation < 88	Yes	0.109	0.196	0.394	0.300
Oxygen saturation < 88	No	0.891	0.804	0.606	0.701
Potassium < 4	No	0.284	0.241	0.195	0.220
Potassium < 4	Yes	0.716	0.759	0.805	0.780
ALT < 13	No	0.961	0.947	0.912	0.939
ALT < 13	Yes	0.039	0.053	0.088	0.061

0 and 1 values are considered as the absence and presence of the clinical variables, respectively. Gray highlighted cells represent the higher condition probability of each variable among four clusters.

Further SIMPLS analysis revealed a very high predictive (*Q*^2^ = 0.63) prediction model to discriminate four clusters based on the 19 most differentiating variables obtained from the primary SIMPLS (Figure 4), showing the significant impact of the above-mentioned variables on each cluster (Table 6). The results demonstrated that the best prediction model was obtained through the combination of patients’ clinical variables and comorbidities with paraclinical variables, while none of them were strong predictors to be separately applied for predicting the mortality. Moreover, the obtained data showed that the categorical paraclinical variables worked better than continuous variables (details are not shown).

### Prediction of ICU admission need using ML-based SIMPLS models

Statistically inspired modification of the partial least square-based prediction models showed moderate predictability (*Q*^2^ = 0.248) for prediction ICU admission need using the SIMPLS-based model on the training set = 1224 and validation set = 519 (Supplementary Figure 1). Although the predictability of the model was similar to that of the mortality model, it suffered from a lower sensitivity (65−70%) than other models. Nonetheless, loss of consciousness, oxygen saturation, heart abnormal findings, CKD, and hypertension were the top five most important predictors among the 12 predictors of the current model (Supplementary Table 3). Interestingly, hypertension, hypotension, and age < 65 were positively correlated with ICU admission (Supplementary Figure 2).

## Discussion

The current study focused on the application of the ML-based statistical method in a clinical setting. The findings indicated that the ML-based SIMPLS model can moderately predict the mortality among COIVD-19 patients using clinical data, comorbidities, and biochemical data. The prediction model set the scene for identifying the most important predictors impacting the mortality of COVID-19 patients. In our study, only 19 variables including 2 demographics, 9 clinical symptoms, 6 comorbidities, and 2 blood chemicals were the potential predictors of the mortality, while many variables were significantly different between non-survivors and survivors. Mortality predictor variables were weighted and ordered based on their importance in the prediction model. Hence, oxygen saturation < 88%, loss of consciousness, CKD, heart abnormal findings, and hypertension were regarded as the top five most important predictors with the highest impact on the model. Nonetheless, the model was less successful without other predictors in the list (Table 1) with less impact coefficient (VIP). The model overfitting was observed with a shorter list of predictors. Our findings using ML-based prediction models showed different patterns of predictors between male and female patients. However, overall oxygen saturation < 88%, loss of consciousness, and CKD were the top three predictors of the mortality among male and female patients. Diabetes and CPD were important comorbidity predictors in male patients, while rheumatic disease and CLD were important comorbidity predictors in female patients. ALT, potassium, urea, creatinine, magnesium, and alkaline phosphatase (ALP) were potential blood chemical predictors in male patients. In female patients, calcium and magnesium were the blood chemical predictors. The LCA-based clustering was able to provide more information about the variables and patterns. Clustering enabled us to characterize the risk factors and easily interpreted them according to 4 clusters of COVID-19 patients. The clustering of patients had additional information to the main prediction model to address the differentiating risk factors of the main model to the subsets of the cohort. Four major clusters revealed the frequency of each differentiating risk factor in different clusters that were not apparent in the main model using two non-survivor and survivor groups. The findings of the clusters can elucidate an important milestone toward a comprehensive understanding of the course of COVID-19 infection that could subsequently be used to optimize disease treatment and patient care. An appropriate clustering method could be a powerful tool for better subgrouping of patients and better grouping of variables in the population. By applying more clusters, future investigations may represent more specific risk factors for each cluster.

One of the main differences of this study in terms of analysis is to apply the ML-based method to develop a model for predicting the mortality using the most important clinical, comorbidities, and chemical factors and determining the variables that affect the performance of the model by applying their weighting in the model. Although the current study was similar to Banoei et al.’s study (27), this study focused on a large cohort of COVID-19 patients, developed two prediction models for males and females, introduced internally multivariate correlation analysis for non-survivors and survivors, and assessed the current SIMPLS-based model using other ML-based models such as SVM, RF, and bootstrap. All the mentioned features differentiated the current study from other similar studies conducted in this regard.

Nonetheless, Banoei et al. (27) reported an ML-based study on 250 confirmed COVID-19 patients with similar predictability for predicting the mortality among a Florida/USA cohort. Captivatingly, CAD, diabetes, age > 65, altered mental status (AMS), oxygen saturation < 88%, and hypertension were the top most important predictors for predicting the mortality in Banoei et al.’s study (27). Excitingly, both studies showed that age 65 years or older was correlated with mortality. Moreover, cardiovascular complications were correlated with an increased mortality in both of these different cohorts. Although diabetes had a negative correlation with the mortality in the basic model in our study, Figure 2 shows that either having or not having diabetes did not have a large impact among all patients for predicting the mortality. Our study further revealed that diabetes was a risk factor for a group of patients with the highest mortality rate.

Age and lower oxygen level have been reported as significant predictors of COVID-19 mortality (5, 28–31). Age has almost been the most significant predictor of the mortality in well-known comorbidities (32). ML-based studies have previously shown that CKD has either a negative (29) or positive (31–33) correlation with the in-hospital mortality among COVID-19 patients. The use of the LASSO approach revealed that the loss of consciousness stood up as the most important predictor of mortality followed by sex, sputum, blood urea nitrogen (BUN), RR, D-Dimer, and age (29). Hypertension has been the second variable among the six important predictors of in-hospital mortality (31).

Many studies have been conducted on developing ML-based models to build and design a model with high accuracy in the field of COVID-19 prognosis or diagnosis (28, 29, 34–43). In the systematic review, Wang et al. (44) examined 78 studies in this regard and reported an accuracy of 70.00−99.92%. Most of these studies were performed in developed countries, and the considered indicators generally included comorbidities, demographic factors, laboratory data and symptoms. Some models also predicted the severity or mortality by considering the genetic indicators or metabolomics (38, 45–48). Image analysis approaches based on deep learning algorithms were also utilized in the field diagnosis and prognosis of COVID-19 patients using CT and radiographic images (11–16). Various indicators have been considered as risk factors for COVID-19 severity and vary from one dataset or country to another (44). The issues that may not have been discussed in previous studies are the importance of each indicator and their relationship. By entering all the factors together and discussing statistical and modeling process details, the issue of importance alone and in relation to other factors was neglected in previous studies. Unlike other studies, the relationship between variables was discussed considering both clinical and statistical aspects in this study. Artificial intelligence studies that use complex statistical analysis to find the relationship between factors and the model design have always been questioned due to the very high mathematical complexity of the medical aspects. In this respect, understanding the relationship between factors and forming different clusters considering these relationships will be more comprehensible medically. In the study conducted by Santosh (49), it is emphasized that a few but major uncertainties may come from multiple sources such as demographics, vulnerability issues originating from underlying comorbidities, hospital settings/capacity, test rates, social distancing issues, and income versus commodities. Therefore, perhaps identifying the best risk factors and their interactions for predicting mortality is of greater significance than merely developing various models with high accuracy.

Unlike previous studies, the correlation and interaction between different risk factors were investigated in this study and shown in Table 3 and Figure 3 as a heat map. The correlation patterns were generally different between survivors and non-survivors. As expected, there were some relationships and positive correlations between some risk factors such as CVD and hypertension, or age and blood pressure. There was a correlation between age and some underlying medical conditions such as cancer or CKD in the survivor group; however, this was not the case for the non-survivor group. The mentioned finding indicated the importance of these conditions regardless of age and suggested that some of these factors independently play an influential role in increasing mortality. This issue has been assessed in another way in Table 6, which divided patients into different mortality categories, providing the opportunity for a better understanding of the existing relationships between factors and the role of each factor in predicting the mortality. For example, clusters 3 and 4 with a mortality rate of 18 and 36%, respectively, correlated with age > 65 though this correlation was generally higher in cluster 3 as compared with cluster 4. In contrast, blood pressure in cluster 4 had a more positive correlation, indicating that blood pressure was not necessarily associated with very elderly patients. Yet, factors such as CVD were more common in cluster 3. Another interesting finding of this study was the significance of CKD and diabetes. CKD, which was found as one of the most crucial factors in predicting the mortality in this study, revealed a relatively independent pattern from that of other factors. Regarding diabetes, although it was stated that it had an overall negative correlation with the mortality, it was considered as one of the influential factors in cluster 4 that played a role in predicting the mortality. This discrepancy observed in this study may be attributed to the high number of diabetic patients (both patients with end-organ damages and patients with appropriate controls) (*N* = 602). Interestingly, there were some differences in terms of some risk factors such as BMI > 24.8, which negatively correlated with the mortality. As shown in Table 3, this factor negatively correlated with most other essential risk factors. In Table 6, the correlation of this factor was high in clusters 1 and 4. The mentioned finding indicated the presence of 2 groups of morbidly obese patients, i.e., the group suffering from other comorbidities and metabolic syndrome and the young group with a healthy status without any other underlying diseases. In general, the main strength of this study was examining the interactions between important risk factors in the prediction of the mortality and revealing the impact of these factors on creating new patterns and new categories for performing mortality analyses as separate models in male and female patients. However, future studies are required to investigate the impact and interaction of different risk factors that were not included in the dataset of this study. Other studies also have reported the better performance of ML algorithms for investigating COVID-19 stratification, mortality risk, and identification of high-risk patients (6, 50).

This study has several limitations. There is not a high certainty to select the appropriate ML method and model for the large dataset. To our knowledge, holistic and concerted care and higher attentiveness must be given to the use of ML-based methods or artificial intelligence in clinical practice by clinical scientists and biologists to interpret the findings. More importantly, the major limitation of the current study is the lack of external validation using an independent validation cohort. Although our prediction model was created using a diverse cohort of multisite study, the validation of the current findings needs to be performed in other populations as the capacity of the health system is not the same among different countries. The prediction model in the current study was obtained with partitioning data to training and validation sets at least once, therefore, future works require repeating the partitioning process several times to the average behavior. Lastly, we cannot control the variables in a retrospective study to establish a predictive model, since the findings may need to be supported using a prospective study. The current data lacks information about the use of steroid treatment among patients which can cause heterogeneity due to immunosuppression’s impact on laboratory values. Moreover, we missed other known biomarkers such as the troponin associated with the severity, particularly COVID-19 pneumonia. Additionally, racial and socioeconomics are important factors to determine the severity of the disease that can be considered as other sources of bias. Regarding the pandemic situation and during the peak of COVID-19 infection, many critically ill patients were forced to turn away due to limited hospital capacity which could be another potential source of bias.

Statistically inspired modification of the partial least square algorithm empirically works with the cross-covariance matrix between response variables and regressor variables in a linear regression approach, hence SIMPLS is very sensitive to outliers. The outlier detection was performed using the explore outlier commands to measure the quantile distribution of the values in a column where the outlier values locate.

## Conclusion

In this study, ML-based models were developed to accurately predict the COVID-19 patients’ mortality. Moreover, the study classified patients into four categories and extracted the main risk factors correlated with mortality and severity in each group. According to the obtained results, low oxygen saturation under 88, loss of consciousness, and CKD were the three leading predictors of mortality. Analyzing the correlation between various factors and assessing their interactions indicated that the relationship pattern of the underlying conditions could differ between male and female patients, which highlights the necessity of conducting further assessments. Moreover, future studies are required in order to externally validate the model and confirm the importance of risk factors generally and in the specified subgroups. In the validation study, the selected features (risk factors) obtained with the training set will be used to predict the probability of mortality in an independent external cohort. AUROC, sensitivity and specificity external validation study will be used to evaluate the model performance.

Machine learning-based statistical methods are different form conventional methods such as logistic regression, Cox regression, generalized additive models, and least-square linear separation methods by providing accurate predictions, enjoying flexibility and scalability, and finding the relationships between variables and internal validity of ML methods using large datasets.

## Data availability statement

The raw data supporting the conclusions of this article will be made available by the authors, without undue reservation.

## Ethics statement

The studies involving human participants were reviewed and approved by the Ethics Committee of Imam Khomeini Hospital Complex- Tehran University of Medical Sciences, Tehran, Iran (No. IR.TUMS.IKHC.REC.1400.040). The patients/participants provided their written informed consent to participate in this study.

## Author contributions

MB contributed to the study concept, statistical analysis interpretation, and drafting of the manuscript. HR contributed to the collection and data cleaning, interpretation and drafting of the manuscript. KZ contributed to providing data, study concept, manuscript interpretation and facilities, and reviewing the manuscript. MS contributed to data collection and cleaning. AN contributed to data collection. FA contributed to data collection and the COVID-19 registry office. SA contributed to data collection, interpretation, and drafting of the manuscript. All authors read and approved the final manuscript.
