# Supplementary material for: Unraveling complex relationships between COVID-19 risk factors using machine learning based models for predicting mortality of hospitalized patients and identification of high-risk group: a large retrospective study
Source: Front Med (Lausanne). 2023 May 4;10:1170331. doi: 10.3389/fmed.2023.1170331 (PMC10192907; doi:10.3389/fmed.2023.1170331)
Supplement: Supplementary file 1 [file Data_Sheet_1.docx]

**Unravelling complex relationships between COVID-19 risk factors using machine learning based models for predicting mortality of hospitalized patients and identification of high-risk group: a large retrospective study.**

Mohammad Mehdi Banoei ^1*^, Haniyeh Rafipour ^2*^, Kazem Zendehdel ^2^, Monireh Seyyed Salehi ^3^, Azin Nahvjou ^3,4^, Farshad Allameh^5^, Saeid Amanpour ^2^

1. Department of Biological Science, University of Calgary, AB, Canada
2. Cancer Biology Research Center, Cancer Institute, Tehran University of Medical Sciences (TUMS), Tehran, Iran.
3. Cancer Research Center, Cancer Institute, Tehran University of Medical Sciences (TUMS), Tehran, Iran.
4. Department of Medical and Surgical Sciences, University of Bologna, 40138 Bologna, Italy
5. Gastroenterology Ward, Imam Khomeini Hospital Complex (IKHC), Tehran University of Medical Sciences, Tehran, Iran

- *These two authors contributed equally to this work and are considered co–first authors.

**Address correspondence to:**

Associated Professor

Deputy of research

Tel: +98 9121325407

Email: Amanpour_S@tums.ac.ir

Cancer Biology Research Center, Cancer Institute, Tehran University of Medical Sciences, Tehran, Iran.

| **Training Set (Men)** | | | | | | | | |
| --- | --- | --- | --- | --- | --- | --- | --- | --- |
| **Method** | **N** | **Entropy R Square** | **Misclassification Rate** | **AUC** | **RASE** | **Generalized R Square** | **Sensitivity** | **Specificity** |
| Bootstrap Forest | 723 | 0.495 | 0.1093 | 0.9673 | 0.26953 | 0.6122 | 67 | 99 |
| Boosted Tree | 723 | 0.4908 | 0.0968 | 0.9482 | 0.26641 | 0.6082 | 57 | 99 |
| Support Vector Machines | 723 | 0.4052 | 0.1176 | 0.8958 | 0.29177 | 0.5215 | 80 | 99 |
| Neural Boosted | 723 | 0.3746 | 0.1342 | 0.8979 | 0.30361 | 0.4888 | 61 | 92 |
| Nominal Logistic | 723 | 0.3356 | 0.1328 | 0.8774 | 0.31349 | 0.4457 | 68 | 95 |
| Decision Tree | 723 | 0.2992 | 0.1549 | 0.8558 | 0.32516 | 0.4039 | 65 | 93 |
| Generalized Regression Lasso | 723 | 0.2766 | 0.1618 | 0.8687 | 0.32812 | 0.3773 | 75 | 97 |
| Fit Stepwise | 723 | 0.2643 | 0.1535 | 0.8544 | 0.33355 | 0.3625 |  |  |
| K Nearest Neighbors | 723 | 0.134 | 0.1881 |  |  |  | 82 | 95 |
|  |  |  |  |  |  |  |  |  |
| **Validation Set (Men)** | | | | | | | | |
| **Method** | **N** | **Entropy R Square** | **Misclassification Rate** | **AUC** | **RASE** | **Generalized R Square** | **Sensitivity** | **Specificity** |
| Fit Stepwise | 308 | 0.26 | 0.1688 | 0.8384 | 0.34947 | 0.3673 |  |  |
| Generalized Regression Lasso | 308 | 0.2532 | 0.1916 | 0.8354 | 0.35121 | 0.3589 | 74 | 96 |
| Neural Boosted | 308 | 0.2507 | 0.1721 | 0.8361 | 0.34873 | 0.3558 | 40 | 92 |
| Decision Tree | 308 | 0.2425 | 0.1981 | 0.8254 | 0.36001 | 0.3456 | 55 | 90 |
| Bootstrap Forest | 308 | 0.2243 | 0.2013 | 0.8211 | 0.36335 | 0.3226 | 76 | 95 |
| Boosted Tree | 308 | 0.1968 | 0.1883 | 0.8017 | 0.36533 | 0.287 | 71 | 95 |
| Nominal Logistic | 308 | 0.1603 | 0.1753 | 0.8014 | 0.36686 | 0.2382 | 55 | 92 |
| Support Vector Machines | 308 | 0.1352 | 0.1818 | 0.8014 | 0.37377 | 0.2033 | 83 | 98 |
| K Nearest Neighbors | 308 | 0.0904 | 0.1981 |  |  |  | 77 | 96 |

**Table S1**. Model screening of prediction mortality among men shows a high AUC and specificity for most ML-based methods such as support vector machine, neural boosted and K Nearest Neighbors.

| **Training Set (Women)** | | | | | | | | |
| --- | --- | --- | --- | --- | --- | --- | --- | --- |
| **Method** | **N** | **Entropy R Square** | **Misclassification Rate** | **AUC** | **RASE** | **Generalized R Square** | **Sensitivity** | **Specificity** |
| Boosted Tree | 501 | 0.4398 | 0.0978 | 0.9298 | 0.26979 | 0.5482 | 70 | 98 |
| Bootstrap Forest | 501 | 0.4394 | 0.1118 | 0.947 | 0.27507 | 0.5478 | 68 | 99 |
| Neural Boosted | 501 | 0.4301 | 0.0978 | 0.9209 | 0.27442 | 0.5382 | 72 | 98 |
| Support Vector Machines | 501 | 0.4203 | 0.0938 | 0.9011 | 0.27 | 0.5281 | 85 | 100 |
| Decision Tree | 501 | 0.3782 | 0.1238 | 0.8861 | 0.29597 | 0.4836 | 68 | 98 |
| Nominal Logistic | 501 | 0.3636 | 0.1098 | 0.8904 | 0.29061 | 0.4677 | 71 | 97 |
| Fit Stepwise | 501 | 0.3634 | 0.1118 | 0.8902 | 0.29065 | 0.4675 |  |  |
| Generalized Regression Lasso | 501 | 0.3585 | 0.1158 | 0.8901 | 0.29203 | 0.4621 | 68 | 97 |
| K Nearest Neighbors | 501 | 0.1154 | 0.1497 |  |  |  | 82 | 97 |
|  |  |  |  |  |  |  |  |  |
| **Validation Set (Women)** | | | | | | | | |
| **Method** | **N** | **Entropy R Square** | **Misclassification Rate** | **AUC** | **RASE** | **Generalized R Square** | **Sensitivity** | **Specificity** |
| Neural Boosted | 211 | 0.3066 | 0.09 | 0.8703 | 0.27378 | 0.3868 | 72 | 99 |
| Generalized Regression Lasso | 211 | 0.2601 | 0.1043 | 0.8477 | 0.28266 | 0.3336 | 72 | 99 |
| Fit Stepwise | 211 | 0.2568 | 0.1043 | 0.8465 | 0.2844 | 0.3297 |  |  |
| Nominal Logistic | 211 | 0.2558 | 0.1043 | 0.8452 | 0.28443 | 0.3285 | 72 | 98 |
| Decision Tree | 211 | 0.245 | 0.1137 | 0.8304 | 0.29347 | 0.3159 | 80 | 97 |
| Bootstrap Forest | 211 | 0.2432 | 0.109 | 0.8367 | 0.29225 | 0.3137 | 80 | 99 |
| Support Vector Machines | 211 | 0.2131 | 0.109 | 0.8069 | 0.28765 | 0.2779 | 96 | 100 |
| Boosted Tree | 211 | 0.195 | 0.1137 | 0.8161 | 0.29181 | 0.2559 | 84 | 98 |
| K Nearest Neighbors | 211 | 0.1353 | 0.109 |  |  |  | 84 | 99 |

**Table S2.** Model screening of prediction mortality among women shows a high AUC and specificity for most ML-based methods such as support vector machine, neural boosted and K Nearest Neighbors.


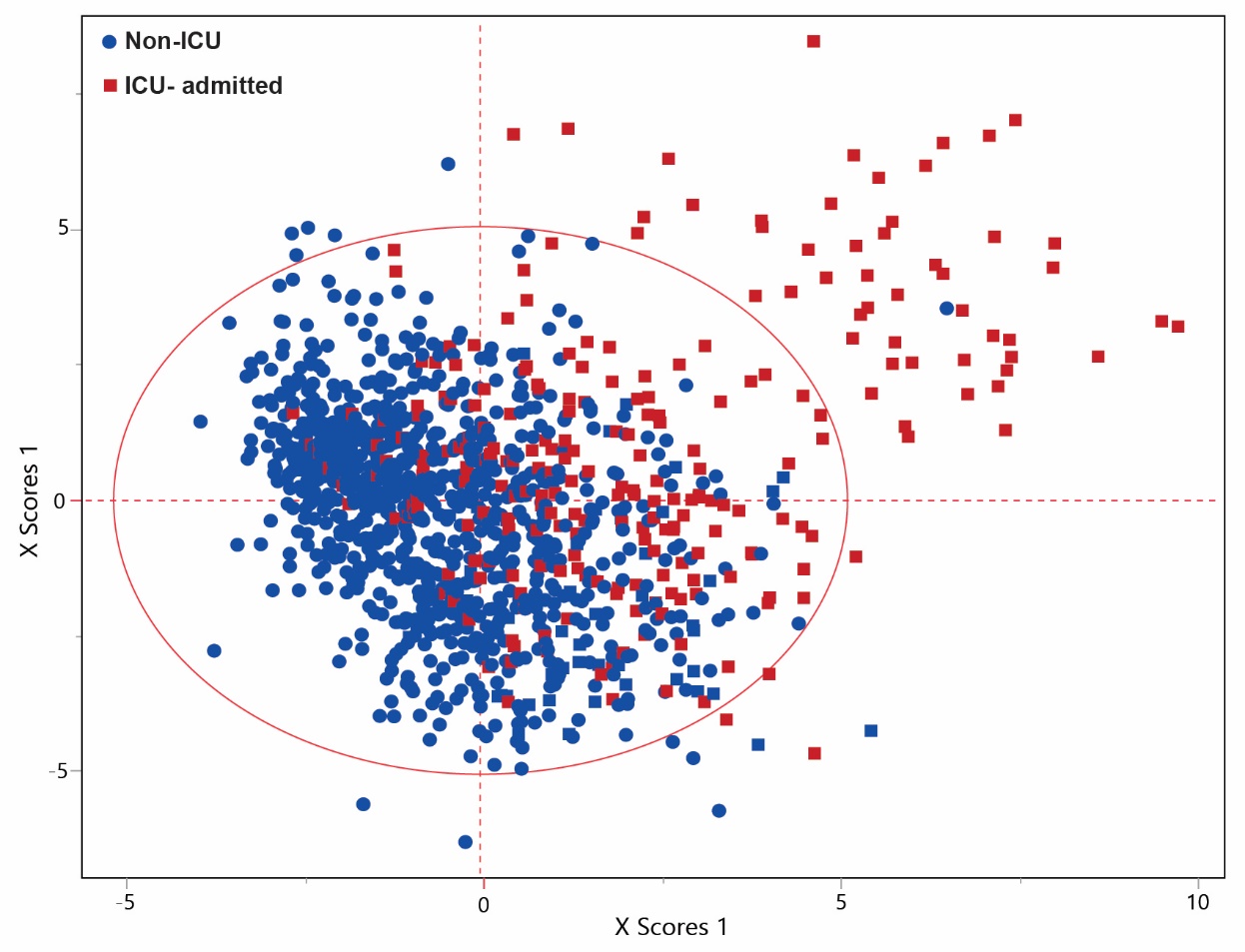


Figure S1. SIMPLS-based scatter plot illustrated the model for the prediction ICU-admission need for a moderate predictability (Q^2^= 0.248)

| **Predictors** | **VIP** |
| --- | --- |
| Loss of Consciousness | 2.01 |
| Oxygen saturation < 88 | 1.23 |
| Heart Abnormal Finding | 1.23 |
| Chronic Kidney Disease | 1.18 |
| Hypertension | 1.13 |
| Cardiovascular Diseases | 0.71 |
| Diabetes | 0.59 |
| Hypotension | 0.56 |
| Age >65 | 0.53 |
| Limb Edema | 0.49 |
| Lymph adenopathy | 0.47 |
| Sputum | 0.46 |

Table S3. The VIP table shows the most differentiating variable to predict ICU admission need. The VIP scores are the own variable score in the best prediction model. The VIP scores were higher >0.8 in the basic model using all variables before variable reduction procedure.


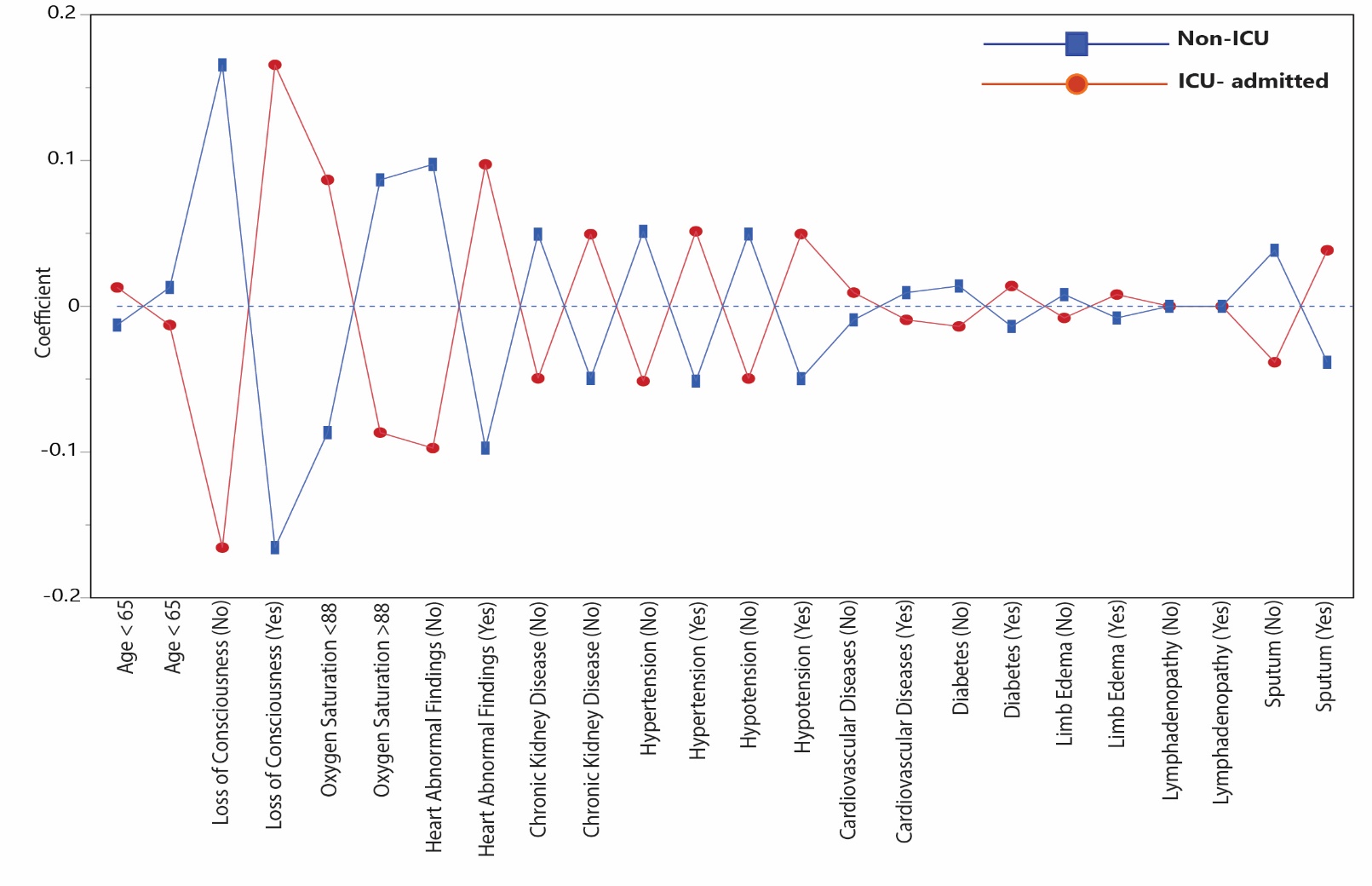


Figure S2. Coefficient plot shows the relative correlation of 12 most differentiating variables to predict ICU admission need. Loss of consciousness, oxygen saturation <88 and heart abnormal findings shows the highest relative correlation with ICU admission need.
